# Quantifying prediction of pathogenicity for within-codon concordance (PM5) using 7541 functional classifications of *BRCA1* and *MSH2* missense variants

**DOI:** 10.1016/j.gim.2021.11.011

**Published:** 2022-03

**Authors:** Lucy Loong, Cankut Cubuk, Subin Choi, Sophie Allen, Beth Torr, Alice Garrett, Chey Loveday, Miranda Durkie, Alison Callaway, George J. Burghel, James Drummond, Rachel Robinson, Ian R. Berry, Andrew Wallace, Diana M. Eccles, Marc Tischkowitz, Sian Ellard, James S. Ware, Helen Hanson, Clare Turnbull, CanVIG-UK, S. Samant, A. Lucassen, A. Znaczko, A. Shaw, A. Ansari, A. Kumar, A. Donaldson, A. Murray, A. Ross, A. Taylor-Beadling, A. Taylor, A. Innes, A. Brady, A. Kulkarni, A.-C. Hogg, A. Ramsay Bowden, A. Hadonou, B. Coad, B. McIldowie, B. Speight, B. DeSouza, B. Mullaney, C. McKenna, C. Brewer, C. Olimpio, C. Clabby, C. Crosby, C. Jenkins, C. Armstrong, C. Bowles, C. Brooks, C. Byrne, C. Maurer, D. Baralle, D. Chubb, D. Stobo, D. Moore, D. O'Sullivan, D. Donnelly, D. Randhawa, D. Halliday, E. Atkinson, E. Baple, E. Rauter, E. Johnston, E. Woodward, E. Maher, E. Sofianopoulou, E. Petrides, F. Lalloo, F. McRonald, F. Pelz, I. Frayling, G. Evans, G. Corbett, G. Rea, H. Clouston, H. Powell, H. Williamson, H. Carley, H.J.W. Thomas, I. Tomlinson, J. Cook, J. Hoyle, J. Tellez, J. Whitworth, J. Williams, J. Murray, J. Campbell, J. Tolmie, J. Field, J. Mason, J. Burn, J. Bruty, J. Callaway, J. Grant, J. Del Rey Jimenez, J. Pagan, J. VanCampen, J. Barwell, K. Monahan, K. Tatton-Brown, K.-R. Ong, K. Murphy, K. Andrews, K. Mokretar, K. Cadoo, K. Smith, K. Baker, K. Brown, K. Reay, K. McKay Bounford, K. Bradshaw, K. Russell, K. Stone, K. Snape, L. Crookes, L. Reed, L. Taggart, L. Yarram, L. Cobbold, L. Walker, L. Walker, L. Hawkes, L. Busby, L. Izatt, L. Kiely, L. Hughes, L. Side, L. Sarkies, K.-L. Greenhalgh, M. Shanmugasundaram, M. Duff, M. Bartlett, M. Watson, M. Owens, M. Bradford, M. Huxley, M. Slean, M. Ryten, M. Smith, M. Ahmed, N. Roberts, C. O'Brien, O. Middleton, P. Tarpey, P. Logan, P. Dean, P. May, P. Brace, R. Tredwell, R. Harrison, R. Hart, R. Kirk, R. Martin, R. Nyanhete, R. Wright, R. Martin, R. Davidson, R. Cleaver, S. Talukdar, S. Butler, J. Sampson, S. Ribeiro, S. Dell, S. Mackenzie, S. Hegarty, S. Albaba, S. McKee, S. Palmer-Smith, S. Heggarty, S. MacParland, S. Greville-Heygate, S. Daniels, S. Prapa, S. Abbs, S. Tennant, S. Hardy, S. MacMahon, T. McVeigh, T. Foo, T. Bedenham, T. Cranston, T. McDevitt, V. Clowes, V. Tripathi, V. McConnell, N. Woodwaer, Y. Wallis, Z. Kemp, G. Mullan, L. Pierson, L. Rainey, C. Joyce, A. Timbs, A.-M. Reuther, B. Frugtniet, B. DeSouza, C. Husher, C. Lawn, C. Corbett, D. Nocera-Jijon, D. Reay, E. Cross, F. Ryan, H. Lindsay, J. Oliver, J. Dring, J. Spiers, J. Harper, K. Ciucias, L. Connolly, M. Tsang, R. Brown, S. Shepherd, S. Begum, S. Daniels, T. Tadiso, T. Linton-Willoughby, H. Heppell, K. Sahan, L. Worrillow, Z. Allen, M. Barlett, C. Watt, M. Hegarty

**Affiliations:** 1 Division of Genetics and Epidemiology, The Institute of Cancer Research, Sutton, United Kingdom; 2 Sheffield Diagnostic Genetics Service, NHS North East and Yorkshire Genomic Laboratory Hub, Sheffield Children's NHS Foundation Trust, Sheffield, United Kingdom; 3 Wessex Regional Genetics Laboratory, Salisbury NHS Foundation Trust, Salisbury, United Kingdom; 4 Human Genetics and Genomic Medicine, Faculty of Medicine, University of Southampton, Southampton, United Kingdom; 5 Manchester Centre for Genomic Medicine and North West Genomic Laboratory Hub, Manchester University NHS Foundation Trust, Manchester, United Kingdom; 6 East Genomic Laboratory Hub, Cambridge University Hospitals Genomic Laboratory, Cambridge University Hospitals NHS Foundation Trust, Cambridge, United Kingdom; 7 North East and Yorkshire Genomic Laboratory Hub, The Leeds Teaching Hospitals NHS Trust, Leeds, United Kingdom; 8 Bristol Genetics Laboratory, Pathology Sciences, Southmead Hospital, North Bristol NHS Trust, Bristol, United Kingdom; 9 Cancer Sciences, Faculty of Medicine, University of Southampton, Southampton, United Kingdom; 10 Department of Medical Genetics, NIHR Research Cambridge Biomedical Research Centre, University of Cambridge, Cambridge, United Kingdom; 11 Department of Molecular Genetics, Royal Devon and Exeter NHS Foundation Trust, Exeter, United Kingdom; 12 National Heart and Lung Institute, Faculty of Medicine, and MRC London Institute of Medical Sciences, Imperial College London, London, United Kingdom; 13 NIHR Royal Brompton Cardiovascular Research Centre, Royal Brompton and Harefield NHS Foundation Trust, London, United Kingdom; 14 Department of Clinical Genetics, St. George’s University Hospitals NHS Foundation Trust, London, United Kingdom; 15 Cancer Genetics Unit, The Royal Marsden NHS Foundation Trust, London, United Kingdom

**Keywords:** ACMG, Classification, Codon, PM5, Variant

## Abstract

**Purpose:**

Conditions and thresholds applied for evidence weighting of within-codon concordance (PM5) for pathogenicity vary widely between laboratories and expert groups. Because of the sparseness of available clinical classifications, there is little evidence for variation in practice.

**Methods:**

We used as a truthset 7541 dichotomous functional classifications of *BRCA1* and *MSH2*, spanning 311 codons of *BRCA1* and 918 codons of *MSH2*, generated from large-scale functional assays that have been shown to correlate excellently with clinical classifications. We assessed PM5 at 5 stringencies with incorporation of 8 in silico tools. For each analysis, we quantified a positive likelihood ratio (pLR, true positive rate/false positive rate), the predictive value of PM5-lookup in ClinVar compared with the functional truthset.

**Results:**

pLR was 16.3 (10.6-24.9) for variants for which there was exactly 1 additional colocated deleterious variant on ClinVar, and the variant under examination was equally or more damaging when analyzed using BLOSUM62. pLR was 71.5 (37.8-135.3) for variants for which there were 2 or more colocated deleterious ClinVar variants, and the variant under examination was equally or more damaging than at least 1 colocated variant when analyzed using BLOSUM62.

**Conclusion:**

These analyses support the graded use of PM5, with potential to use it at higher evidence weighting where more stringent criteria are met.

## Introduction

### Variant interpretation

Sequence analysis of constitutional DNA has informed diagnosis and prediction of human Mendelian diseases for >3 decades. Correct identification of the causative pathogenic variant is necessary if prediction of the clinical course of disease, implementation of measures for prevention, and early detection are to be effective. Through technological advances, clinical genome sequencing is now routine. In an average human, this typically reveals an excess of 4 million variants compared with a reference human genome.^1^ To reduce erroneous assignment of variants as pathogenic, there have been concerted efforts within the clinical laboratory community to produce consensus frameworks for variant interpretation, such as that of the American College of Medical Genetics and Genomics/Association of Molecular Pathology (ACMG/AMP).^2^ This framework comprises 5 levels of variant classification based on weighted summing of different lines of evidence such as clinical case series, segregation data, phenotypic specificity, and laboratory (functional) assays.^2^ In parallel, ClinVar has been established as a freely available public repository for classifications, hosted by the National Center for Biotechnology Information.^3^ Its ranking system reflects the robustness of the classification; a 3-star classification is only awarded if the classification has been awarded by (1) a ClinVar recognized expert panel or (2) a ClinGen Variant Curation Expert Panel (VCEP), an expert panel providing Food and Drug Administration–recognized variant interpretation using ACMG/AMP evidence codes and specifications.^4^

### Within-codon concordance of pathogenic variants (PM5)

In many regions of a gene, variants are well tolerated without discernible effect on protein function. However, there are residues at which substitution of even a seemingly similar amino acid will have a dramatic effect on the structure and/or function of the protein. It is thus reasonable to hypothesize that a codon at which other previously-encountered missense substitutions have been shown to be pathogenic encodes an amino acid that is structurally and/or functionally important. Hence, a novel missense substitution identified at that codon is relatively more likely than average to also be pathogenic. Conversely, it is reasonable to hypothesize that at a codon at which previously-encountered missense substitutions have all been shown to be benign, the amino acid is overall likely to be nonessential to protein structure and function. A novel missense substitution at that codon is thus overall more likely than average to be benign. This is a well-cemented axiom in interpretation of novel sequence variants, and is ascribed moderate evidence (evidence item PM5) in the 2015 ACMG/AMP variant interpretation framework.^2^ However, specifications by different VCEPs of rules around the usage of PM5 vary widely, eg, prescribing different evidence weighting, incorporating different in silico tools, allowing application across paralogous genes, or prohibiting the use of PM5 altogether.^5^, ^6^, ^7^, ^8^, ^9^. For most genes, clinical variant classifications are typically only available for a very sparse set of variants that are potentially biased toward particular regions and codons. This means that the validation and therefore justification of the different VCEP PM5 specifications to date have been limited.^10^ PM5 is widely used across laboratories for variant interpretation. Correct calibration of evidence weighting and combination is essential to ensure that our final classifications of variants are accurate.

### In silico predictions of effect of missense variants

Amino acids vary in composition, polarity, and molecular volume. More dramatic differences between wild-type and variant amino acids in these physiochemical parameters are more likely to alter the structure and thus function of the protein. In 1974, Grantham^11^ proposed the Grantham Difference as a score for quantifying this physiochemical difference between amino acids. Following this, amino acid substitution scoring matrices such as the PAM250 or BLOSUM scores incorporated pairwise comparisons of physiochemical characteristics alongside evolutionary substitution frequencies.^12^ In subsequent tools, such as Align-GVGD, protein multiple sequence alignments were also incorporated to capture the essentiality of the wild-type amino acid as well as the physiochemical magnitude of the substitution.^13^ Numerous subsequent in silico tools have emerged, which variously predict the severity of the effect of a missense variant using these and other elements, such as predicted disruption to 3-dimensional protein structure, information about protein domains, clinical annotations, and population allele frequency data. Newer meta tools such as REVEL and Meta-SNP use machine learning across multiple tools to optimize predictive performance.^14^

### Large-scale functional assays of cancer susceptibility genes

The deleteriousness of a missense variant can also be quantified by measuring, in an ex vivo cellular construct, its effect on a relevant cellular function. Early functional assays were laborious and thus low throughput; typically only a selected handful of clinically-observed variants would be included. After the advances in gene editing technology and multiplex assay design, high throughput saturation genome editing experiments have made it possible to assay simultaneously many thousands of variants via robust systematic methodologies called multiplex assays of variant effect (MAVEs).^15^ For some MAVEs for which sufficient clinical classifications exist for comparison, high concordance has been shown with discrepancies highlighting potential clinical misclassifications.^16^

MAVEs provide unbiased systematic functional classifications of (nearly) every missense variant that can arise by single base substitution at a codon. These data sets therefore offer a novel opportunity for evaluation of PM5. To explore this further, we selected 2 MAVEs (for *BRCA1* and *MSH2*) for which (1) adequate validation had been possible because multiple ClinVar Expert Panel 3-star clinical classifications of benign and pathogenic variants are available on ClinVar, (2) the MAVE has not yet been widely used by the Expert Panels for generation of these clinical classifications, and (3) high concordance of MAVE-functional classifications with clinical classifications has been shown. In this study, we explored for these 2 genes the predictive strength of PM5, quantified as a likelihood ratio.

## Materials and Methods

### Functional classifications for *BRCA1* and *MSH2* variants

For *BRCA1,* we used data on 3893 single-nucleotide variants in the 13 exons encompassing the RING finger motif and BRCT (BRCA1 C-terminal) functional domain, generated by Findlay et al^16^ using saturation mutagenesis. Findlay et al^16^ assessed variant-*BRCA1* function using an assay of cellular fitness of HAP1 cells (a near-haploid cancer cell line). For *MSH2*, we used data on 5212 MSH2 amino acid substitutions that corresponded to 5734 nonsynonymous single-nucleotide variants, generated by Jia et al^17^ using saturation mutagenesis. Jia et al^17^ assessed variant-*MSH2* function using an assay of HAP1 cellular survival after treatment with 6-thioguanine, which is selectively toxic to mismatch repair proficient cells as it induces lesions unrepairable by the mismatch repair machinery (Supplemental Table 1). Data from RNA sequencing was only available for *BRCA1*, and thus, for parity this was not included in the main analysis. Each functional truthset was curated to include only missense variants. Synonymous, nonsense, and initiation codon variants were excluded. The potentially spliceogenic exonic variants at the 2 bases flanking the intron–exon boundary were also excluded (hereafter called para-splice-site variants). Variants were described in accordance with Human Genome Variation Society nomenclature for GRCh37 transcripts ENST00000357654 (*BRCA1*) and ENST00000233146 (*MSH2*). The calculation of PM5 positive likelihood ratios (pLRs) requires dichotomous functional classifications, and therefore, variants with intermediate assay activity were excluded. The remainder were included as classified in their original publications as either deleterious (DEL) or tolerated (TOL).

### Clinical classifications for *BRCA1* and *MSH2* variants

We assembled available ClinVar classifications for missense variants in the corresponding codons of *BRCA1/MSH2*, again excluding para-splice-site variants. Variants with a ClinVar classification of ≥1-star rating of pathogenic/likely pathogenic (P/LP) or benign/likely benign (B/LB), were assigned to dichotomous clinical classification groups: ClinVar DEL or ClinVar TOL. The clinical classification was designated as missing for variants for which there was no classification, a classification of uncertain significance, or conflicting interpretations of pathogenicity. The concordance between clinical and functional classifications is shown in Supplemental Table 2.

### In silico annotations for *BRCA1* and *MSH2* variants

For each variant we retrieved predictions for selected in silico tools. BLOSUM45, BLOSUM62, BLOSUM80, Grantham Score, and Align-GVGD were selected because they specifically reflect the physiochemical difference between the wild-type and variant amino acid.^11^, ^12^, ^13^^,^^18^^,^^19^ REVEL, Meta-SNP, and CADD were selected because these tools are widely-used clinically and/or assessed as high-performing.^14^^,^^20^, ^21^, ^22^ In silico scores were retrieved using Annovar (dbnsfp33a database), Alamut-HT, the Meta-SNP, and REVEL webservers.^14^^,^^20^^,^^23^^,^^24^

### Generation and evaluation of PM5 predictions

**Figure 1:**
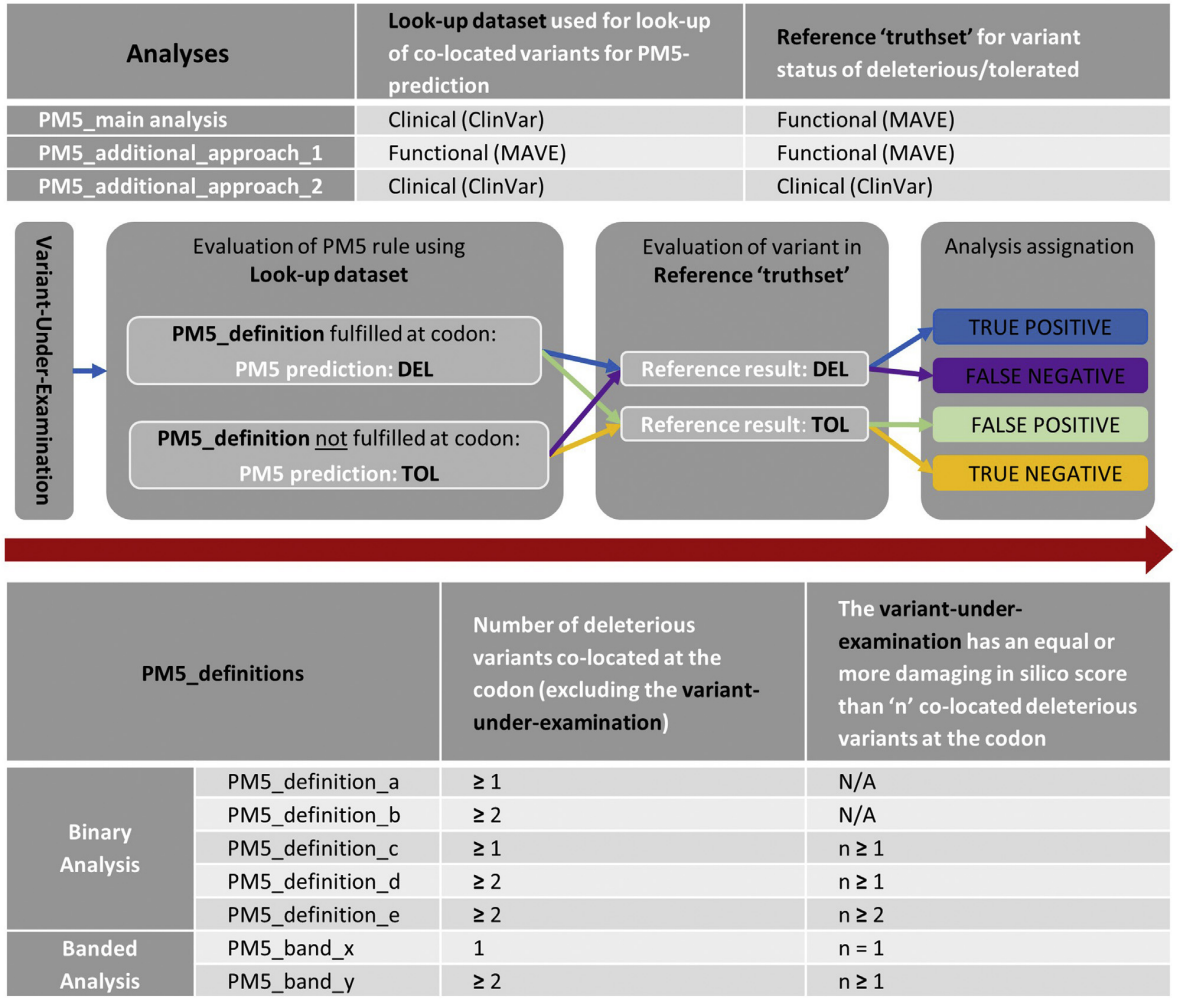
**Schematic of PM5 analyses comparing prediction (lookup of colocated variants in the lookup data set) with a reference truthset**. Combinations of lookup data set and reference truthset for each analysis approach (top). Assignation of true positive, true negative, false positive, and false negative (middle). Binary PM5 definitions of increasing stringency (a-e) and nonoverlapping banded PM5 definitions (x, y) (bottom). DEL, deleterious; MAVE, multiplex assay of variant effect; TOL, tolerated.

We considered 5 definitions of PM5 (PM5-definitions a-e) of varying stringency relating to (1) number of DEL variants colocated at the codon of interest (excluding the variant under examination) and (2) whether the variant under examination had an equally or more damaging in silico prediction than the colocated DEL variants, reflecting the variation in existing VCEP criteria (Figure 1). PM5-definition_a is the least stringent, whereas PM5-definition_e is the most stringent, mandating the greatest number of colocated DEL variants and requirement for more damaging performance on in silico tools. Our primary approach was PM5-lookup using clinical classifications (classifications from ClinVar, *n* = 199) to make the PM5 prediction, referenced against a truthset of dichotomous functional classifications (*BRCA1*/*MSH2* MAVEs, *n* = 7541). All variants for which a dichotomous functional classification was available were assessed; missense variants were not included in the analysis if there was no MAVE data or the MAVE output was intermediate.

If lookup for the variant under examination in ClinVar met the stated conditions of the PM5 definition, the PM5 prediction was DEL. If the variant under examination did not meet the stated conditions, the PM5 prediction was TOL. The PM5 prediction was then compared with the variant’s classification in the MAVE reference truthset. Assignment of true positive (TP) was made for a variant for which PM5 prediction in ClinVar was DEL and classification in the MAVE reference truthset was also DEL, and assignment of true negative (TN) was made when PM5 prediction in ClinVar was TOL and classification was TOL in the MAVE reference truthset. The variant was assigned false positive (FP) when the ClinVar PM5 prediction was DEL but the classification in the MAVE reference truthset was TOL. The variant was assigned false negative (FN) when the ClinVar PM5 prediction was TOL but the classification in the in the MAVE reference truthset was DEL (Figure 1, Supplemental Table 3).

We repeated these analyses first using the MAVE-functional classifications for both the PM5-lookup and reference truthset (Additional_Approach_1) and second using ClinVar clinical classifications for both the PM5-lookup and reference truthset (Additional_Approach_2) (Figure 1).

For each analysis, we quantified PM5 pLRs, ie, the TP rate/FP rate ([TP/(TP+FN)]/[FP/(TN+FP)]) (Table 1, Supplemental Tables 4 and 5). We applied a Haldane-Anscombe correction (0.5 added to each cell) to (1) allow generation of PM5 pLRs where there are 0 value cells, and (2) to add a conservative correction. PM5 negative likelihood ratios were also calculated ([TN/(TN+FP)]/[FN/(FN+TP)]) (Supplemental Table 6).

**Table 1:** Positive LR for different definitions of PM5 for binary analyses of data for (1) *BRCA1* and *MSH2* combined, (2) *BRCA1*, and (3) *MSH2*

PM5-Definition	Tool	*BRCA1* + *MSH2*					*BRCA1*					*MSH2*				
		TP	FN	FP	TN	Positive LR	TP	FN	FP	TN	Positive LR	TP	FN	FP	TN	Positive LR
PM5_a ≥ 1 deleterious reference variants at codon		245	556	244	6496	**8.4 (7.2-9.9)**	161	233	97	1316	**5.9 (4.7-7.4)**	84	323	147	5180	**7.5 (5.8-9.6)**
PM5_b ≥ 2 deleterious reference variants at codon		111	690	59	6681	**15.8 (11.6-21.4)**	84	310	29	1384	**10.3 (6.8-15.4)**	27	380	30	5297	**11.8 (7.1-19.5)**
PM5_c ≥ 1 deleterious reference variant at codon; variant under examination has an equal or more damaging in silico score than ≥1 colocated variant	REVEL	122	679	52	6688	**19.6 (14.3-26.9)**	84	310	15	1398	**19.5 (11.5-33.2)**	38	369	37	5290	**13.4 (8.6-20.8)**
	Meta-SNP	145	656	29	6711	**41.5 (28.1-61.2)**	99	295	9	1404	**37.5 (19.5-72.3)**	46	361	20	5307	**29.6 (17.8-49.3)**
	CADD	154	647	77	6663	**16.8 (12.9-21.8)**	109	285	37	1376	**10.5 (7.3-14.9)**	45	362	40	5287	**14.7 (9.7-22.1)**
	Grantham Score	142	659	50	6690	**23.7 (17.4-32.4)**	90	304	20	1393	**15.8 (9.9-25.2)**	52	355	30	5297	**22.5 (14.6-34.7)**
	aGVGD	141	660	54	6686	**21.8 (16.1-29.6)**	90	304	14	1399	**22.3 (13.0-38.5)**	51	356	40	5287	**16.6 (11.1-24.8)**
	BLOSUM45	133	668	40	6700	**27.7 (19.6-39.1)**	91	303	12	1401	**26.2 (14.7-46.8)**	42	365	28	5299	**19.5 (12.2-31.0)**
	BLOSUM62	139	662	42	6698	**27.6 (19.7-38.6)**	93	301	12	1401	**26.8 (15.0-47.8)**	46	361	30	5297	**19.9 (12.8-31.1)**
	BLOSUM80	133	668	43	6697	**25.8 (18.5-36.0)**	87	307	12	1401	**25.1 (14.0-44.8)**	46	361	31	5296	**19.3 (12.4-30.0)**
PM5_d ≥ 2 deleterious reference variants at codon; variant under examination has an equal or more damaging in silico score than ≥1 colocated variant	REVEL	69	732	15	6725	**37.7 (21.8-65.0)**	53	341	4	1409	**42.6 (16.4-110.7)**	16	391	11	5316	**18.7 (8.9-39.5)**
	Meta-SNP	81	720	6	6734	**105.4 (47.6-233.5)**	62	332	4	1409	**49.7 (19.2-128.6)**	19	388	2	5325	**101.9 (27.4-378.6)**
	CADD	86	715	28	6712	**25.5 (16.8-38.7)**	68	326	16	1397	**14.9 (8.8-25.1)**	18	389	12	5315	**19.3 (9.5-39.3)**
	Grantham Score	73	728	5	6735	**112.3 (47.4-266.3)**	52	342	2	1411	**75.2 (21.2-266.0)**	21	386	3	5324	**80.2 (26.0-247.1)**
	aGVGD	72	729	19	6721	**31.3 (19.1-51.2)**	52	342	2	1411	**75.2 (21.2-266.0)**	20	387	17	5310	**15.3 (8.1-28.7)**
	BLOSUM45	77	724	9	6731	**68.6 (35.1-134.0)**	58	336	4	1409	**46.5 (18.0-120.6)**	19	388	5	5322	**46.3 (18.1-118.6)**
	BLOSUM62	83	718	10	6730	**66.8 (35.3-126.5)**	61	333	4	1409	**48.9 (18.9-126.6)**	22	385	6	5321	**45.2 (19.0-107.6)**
	BLOSUM80	79	722	9	6731	**70.3 (36.0-137.3)**	59	335	3	1410	**60.9 (20.8-177.8)**	20	387	6	5321	**41.2 (17.1-98.9)**
PM5_e ≥ 2 deleterious reference variants at codon; variant under examination has an equal or more damaging in silico score than ≥ 2 colocated variants	REVEL	42	759	7	6733	**47.6 (22.0-103.2)**	34	360	1	1412	**82.3 (16.1-420.6)**	8	399	6	5321	**17.1 (6.2-47.2)**
	Meta-SNP	49	752	3	6737	**118.9 (40.3-350.6)**	36	358	2	1411	**52.3 (14.6-187.3)**	13	394	1	5326	**117.5 (21.8-633.1)**
	CADD	52	749	17	6723	**25.2 (14.8-43.1)**	43	351	11	1402	**13.5 (7.1-25.7)**	9	398	6	5321	**19.1 (7.1-51.5)**
	Grantham Score	44	757	1	6739	**249.4 (49.1-1,266.8)**	34	360	0	1413	**247.0 (15.2-4,019.8)**	10	397	1	5326	**91.4 (16.6-504.3)**
	aGVGD	43	758	3	6737	**104.5 (35.2-309.6)**	34	360	0	1413	**247.0 (15.2-4,019.8)**	9	398	3	5324	**35.4 (10.5-120.2)**
	BLOSUM45	49	752	4	6736	**92.5 (35.3-242.0)**	41	353	0	1413	**297.1 (18.3-4,819.2)**	8	399	4	5323	**24.7 (7.9-77.0)**
	BLOSUM62	43	758	5	6735	**66.5 (27.5-160.9)**	37	357	0	1413	**268.5 (16.5-4,362.4)**	6	401	5	5322	**15.4 (5.0-47.8)**
	BLOSUM80	46	755	6	6734	**60.1 (26.6-136.2)**	39	355	1	1412	**94.3 (18.5-479.5)**	7	400	5	5322	**17.8 (6.0-53.3)**

Positive likelihood ratios and 95% confidence intervals are shown in bold.

*FN*, false negative; *FP*, false positive; *LR*, likelihood ratio; *TN*, true negative; *TP*, true positive.

In these binary analyses, variants that meet a more stringent PM5-definition were included in the analyses of less stringent definitions (eg, variants attaining PM5-definition_e necessarily also attain PM5-definition_d). To advance beyond this, we performed a banded analysis in which PM5-definitions were nonoverlapping (exclusive) and compared with a reference baseline band. PM5_band_x was defined as there being only 1 colocated DEL variant compared with which the variant under examination had an equal or more damaging in silico score. PM5_band_y was defined as there being 2 or more colocated DEL variants compared with which the variant under examination had an equal or more damaging in silico score than at least 1 colocated variant. The baseline_band comprised all variants not meeting the criteria for PM5_band_x or PM5_band_y (Table 2, Supplemental Tables 7 and 8).

**Table 2:** Positive LRs for nonoverlapping bands for PM5 for (1) *BRCA1* and *MSH2* combined, (2) *BRCA1*, and (3) *MSH2*

PM5-Definition-Band	Tool	*BRCA1* + *MSH2*					*BRCA1*					*MSH2*				
		TP	FN	FP	TN	Positive LR	TP	FN	FP	TN	Positive LR	TP	FN	FP	TN	Positive LR
PM5_baseline_band: variants not attaining criteria for PM5_band_x or PM5_band_y																
PM5_band_x) Exactly 1 deleterious colocated variant at codon; variant under examination has an equal or more damaging in silico score than colocated variant; comparison with baseline variant set	Revel	53	679	37	6688	**13.1 (8.7-19.7)**	31	310	11	1398	**11.3 (5.8-22.0)**	22	369	26	5290	**11.5 (6.6-20.0)**
	Meta-SNP	64	656	23	6711	**25.6 (16.1-40.9)**	37	295	5	1404	**28.9 (11.9-70.1)**	27	361	18	5307	**20.4 (11.4-36.4)**
	CADD	68	647	49	6663	**13.0 (9.1-18.5)**	41	285	21	1376	**8.3 (5.0-13.7)**	27	362	28	5287	**13.2 (7.9-22.0)**
	Grantham Score	69	659	45	6690	**14.1 (9.8-20.3)**	38	304	18	1393	**8.6 (5.0-14.7)**	31	355	27	5297	**15.8 (9.6-26.0)**
	aGVGD	69	660	35	6686	**18.0 (12.1-26.8)**	38	304	12	1399	**12.7 (6.8-23.7)**	31	356	23	5287	**18.3 (10.9-31.0)**
	BLOSUM45	56	668	31	6700	**16.7 (10.8-25.6)**	33	303	8	1401	**16.5 (7.8-34.7)**	23	365	23	5299	**13.7 (7.8-24.0)**
	BLOSUM62	56	662	32	6698	**16.3 (10.6-24.9)**	32	301	8	1401	**16.1 (7.7-34.0)**	24	361	24	5297	**13.8 (8.0-23.9)**
	BLOSUM80	54	668	34	6697	**14.7 (9.7-22.4)**	28	307	9	1401	**12.6 (6.1-26.0)**	26	361	25	5296	**14.3 (8.4-24.3)**
PM5_band_y) ≥2 deleterious colocated variants at codon; variant under examination has an equal or more damaging in silico score than ≥1 colocated variant; comparison with baseline variant set	Revel	69	679	15	6688	**40.1 (23.3-69.2)**	53	310	4	1398	**45.8 (17.6-119.1)**	16	369	11	5290	**19.7 (9.3-41.5)**
	Meta-SNP	81	656	6	6711	**114.1 (51.5-252.8)**	62	295	4	1404	**54.7 (21.1-141.3)**	19	361	2	5307	**108.7 (29.3-403.9)**
	CADD	86	647	28	6663	**27.7 (18.2-42.0)**	68	285	16	1376	**16.3 (9.7-27.6)**	18	362	12	5287	**20.6 (10.1-41.9)**
	Grantham Score	86	647	28	6663	**27.7 (18.2-42.0)**	52	304	2	1393	**82.1 (23.2-290.5)**	21	355	3	5297	**86.4 (28.0-266.0)**
	aGVGD	72	660	19	6686	**34.0 (20.8-55.8)**	52	304	2	1399	**82.5 (23.3-291.7)**	20	356	17	5287	**16.5 (8.8-30.9)**
	BLOSUM45	77	668	9	6700	**73.4 (37.6-143.3)**	58	303	4	1401	**50.5 (19.5-130.8)**	19	365	5	5299	**48.9 (19.1-125.1)**
	BLOSUM62	83	662	10	6698	**71.5 (37.8-135.3)**	61	301	4	1401	**52.9 (20.5-136.9)**	22	361	6	5297	**47.8 (20.1-113.7)**
	BLOSUM80	79	668	9	6697	**75.0 (38.5-146.4)**	59	307	3	1401	**65.1 (22.3-190.1)**	20	361	6	5296	**43.8 (18.2-105.1)**

Positive likelihood ratios and 95% confidence intervals are shown in bold.

*FN*, false negative; *FP*, false positive; *LR*, likelihood ratio; *TN*, true negative; *TP*, true positive.

To examine the effect of occult midexonic spliceogenic base substitutions (excluding the para-splice-site variants), we used quantitative RNA sequencing data available for the *BRCA1* variants.^16^ We conducted the full PM5 analyses including and then excluding these 31 midexonic variants for which the RNA level was intermediate (17 variants [14 DEL/3 TOL]) or depleted (14 variants [all DEL]) (Supplemental Table 9).

## Results

**Figure 2:**
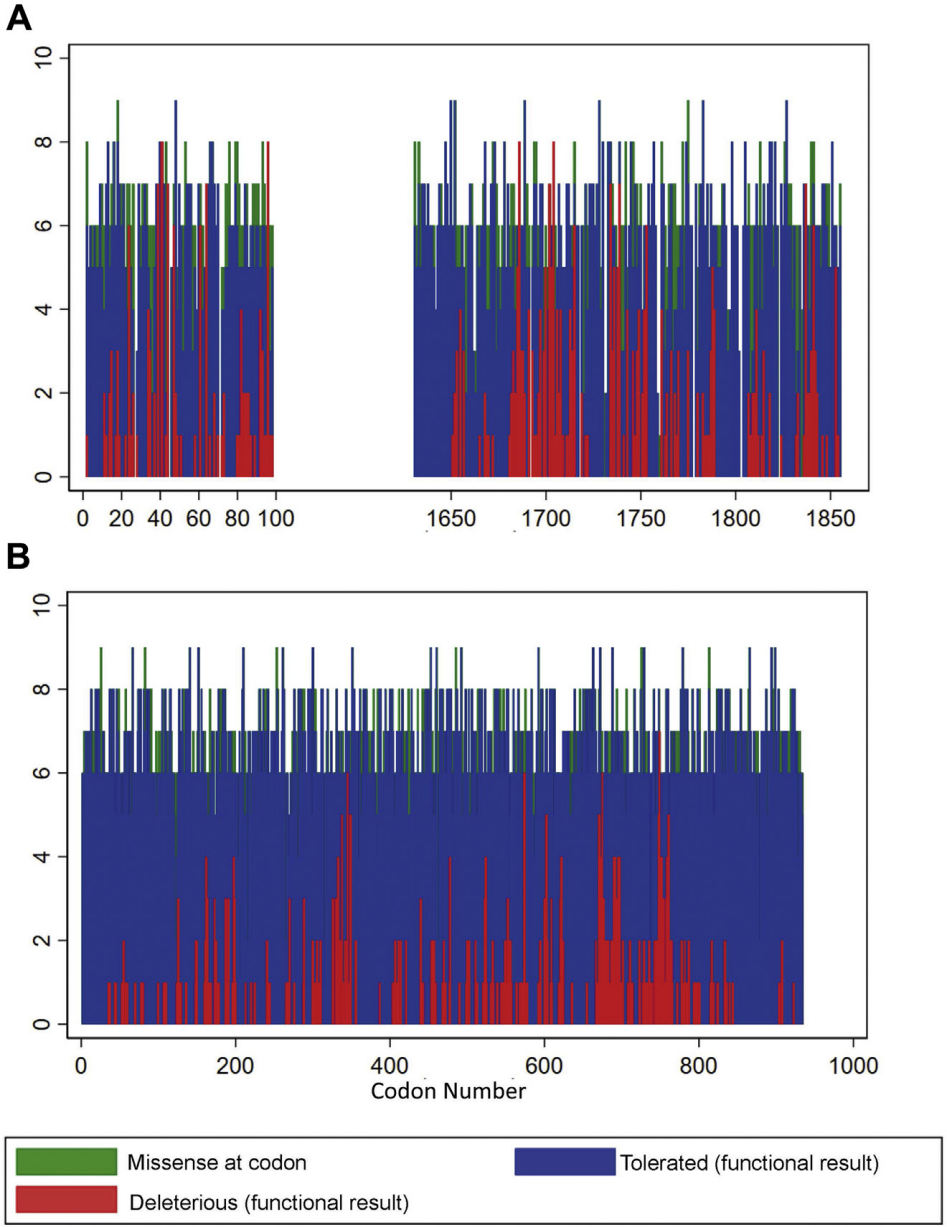
**Distribution of assay results by codon.** By codon, number of multiplex assay of variant effect (MAVE)-deleterious missense variants (red), number of MAVE-tolerated missense variants (blue), and number of eligible missense (green) for (A) *BRCA1* and (B) *MSH2*.

**Figure 3:**
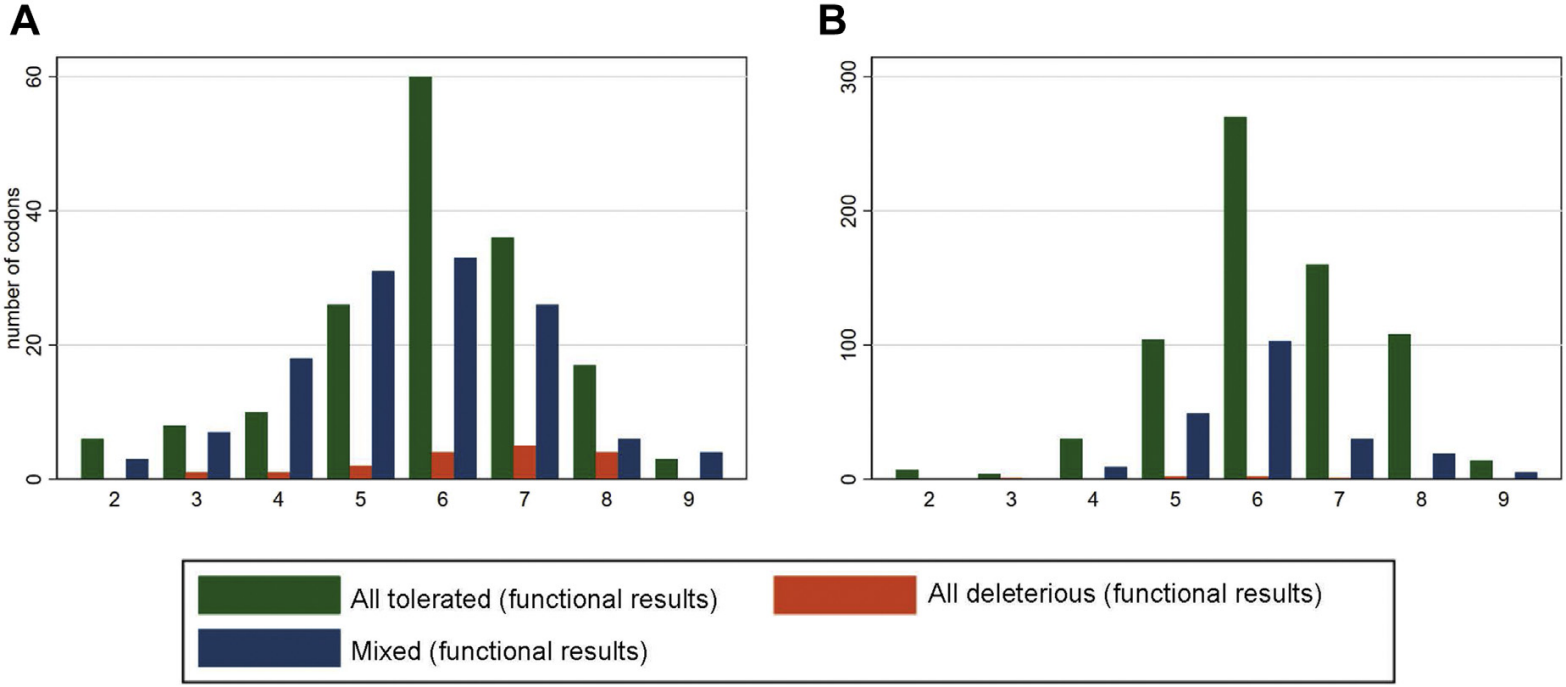
**Distribution of codon typesin *BRCA1* and *MSH2.*** Number of codons which are MAVE-deleterious only (red), MAVE-tolerated only (green), and MAVE-mixed (blue) against the total number of missense variants at the codon for which there is dichotomous assay data (x-axis) for (A) *BRCA1* and (B) *MSH2*.

Excluding ineligible variants and codons, across the 311 codons spanning the RING domain (amino acids 1-98) and BRCT domain (amino acids 1631-1855) of *BRCA1*, dichotomized functional classifications were available for 1807 missense variants (1413 assay-TOL/394 assay-DEL) (Figure 2A), distributed as 17 DEL-only codons, 128 mixed codons, and 166 TOL-only codons (Figure 3A). Dichotomized ClinVar clinical classifications were available for 111 variants (22 B/LB and 89 P/LP).

For MSH2, across the 918 codons studied, dichotomized functional classifications were available on 5734 missense variants (5327 assay-TOL/407 assay-DEL) (Figure 2B), distributed as 6 DEL-only codons, 215 mixed codons, and 697 TOL-only codons (Figure 3B). Dichotomized ClinVar classifications were available for 88 variants (28 B/LB and 60 P/LP).

In total, 7541 variants were analyzed for each of the 5 PM5 definitions (a-e). Overall, PM5 pLRs were higher when the PM5-definition was of higher stringency, eg, e > d > b. Values were broadly similar for *BRCA1* and *MSH2* (Table 1)*.* Combining data from the 2 genes, the PM5 pLR was 8.4 (7.2-9.9) for PM5-definition_a (variants for which there are 1 or more colocated DEL variants at the codon) and 15.8 (11.6-21.4) for PM5-definition_b (variants for which there are 2 or more colocated DEL variants at the codon).

These PM5 pLR increased with application of the stipulation that the variant under examination should be predicted to be more damaging than 1 or more of the reference variants (PM5-definitions c-e) for all 8 tools examined. For example, the PM5 pLR increased to 27.6 (19.7-38.6) for PM5-definition_c (where there was 1 or more colocated DEL variants at the codon and the variant under examination was equally or more damaging using BLOSUM62 than at least 1 colocated DEL variant) and to 66.5 (27.5-160.9) for PM5-definition_e (2 or more colocated DEL variants at the codon and the variant under examination is more or equally damaging using BLOSUM62 than 2 or more of colocated DEL variants).

In the banded analyses of nonoverlapping PM5 definitions compared with a common baseline group, PM5 pLRs were 16.3 (10.6-24.9) for variants attaining standard x (exactly 1 colocated DEL variant; variant under examination equally or more damaging using BLOSUM62) and 71.5 (37.8-135.3) for variants attaining standard y (2 or more colocated DEL variants; variant under examination equally or more damaging using BLOSUM62 than at least 1 colocated DEL variant) (Table 2).

The PM5 pLRs were moderately lower when we used MAVE data both for the PM5-lookup and for the reference truthset (Supplemental Tables 4 and 7). When using ClinVar data for the reference truthset and the PM5-lookup (*n* = 199), because of smaller numbers, the PM5 pLRs exhibited less stable patterns and had wider confidence intervals; at higher stringencies of PM5-definition both the TP rate and FP rate were very low using the ClinVar data (Supplemental Tables 5 and 8).

Exclusion, in addition to para-splice-site variants, of the 31 potentially spliceogenic exonic variants from the *BRCA1* analysis had negligible effect on the PM5 pLRs (Supplemental Table 9).

## Discussion

In these analyses we sought to quantify how often the realworld approach of PM5 is correct. We generated PM5 predictions by lookup in ClinVar of clinically classified colocated DEL (pathogenic) variants. However, we referenced these predictions against a comprehensive, unbiased truthset of functional classifications available for (nearly) every putative variant under examination. We conducted our analyses using *BRCA1* and *MSH2*, which are well-established cancer susceptibility genes for which there have been high volumes of clinical testing and long-established expert groups for clinical variant interpretation.^25^^,^^26^ Although the number of *BRCA1*/*MSH2* missense variants for which dichotomized classifications are available in ClinVar is still small compared with the total number of potential missense variants, this number is far greater than for most other genes. For other genes there typically will be fewer clinical classifications of DEL (pathogenic) variants available to generate TP PM5-calls, meaning that for a greater proportion of genuinely DEL (pathogenic) variants under examination, PM5 will not be attainable. Accordingly, for genes with more sparse clinical classifications, the PM5 pLR estimates presented here are likely to be conservative.

For *MSH2* the described functional domains were distributed across the length of the gene, and no clustering of DEL variants was evident in the MAVE data. By contrast, for *BRCA1*, MAVE data were only available for the RING and BRCT domains owing to established doctrine that there are no DEL (pathogenic) missense variants located outside of these domains. The likelihood ratios were higher for *MSH2* than for *BRCA1* for basic definitions of PM5 (definitions a and b), although there was some variability when in silico predictions were incorporated into the definitions. For *MSH2*, in total, 407 of 5734 (7.1%) missense variants were MAVE-DEL, whereas 697 of 918 (75.9%) codons harbored only MAVE-TOL variants (Figure 3B). By contrast, for *BRCA1*, 394 of 1807 (21.8%) missense variants were MAVE-DEL, whereas 166 of 311 (53.4%) codons harbored only MAVE-TOL variants (Figure 3A). There are 1533 intervening *BRCA1* codons not covered by the Findlay et al^16^ MAVE. If, as presumed, those codons are less important to protein structure and function, single-nucleotide variation at those codons would be largely MAVE TOL. This would increase the TN rate, and thus, the prediction would be that inclusion of a full *BRCA1* variant set would result in increased PM5 pLRs.

It might be anticipated that variants for which ClinVar clinical classifications are available would be a nonrandom sample of all DEL variants, potentially biased toward variants for which richer clinical data may be available and toward recognized hot spots. Of *BRCA1* P/LP variants in ClinVar, 62 of 89 (70%) had a colocated P/LP variant in ClinVar at that codon. For *BRCA1* MAVE-DEL variants, 342 of 394 (87%) had a colocated MAVE-DEL variant at that codon. For *MSH2*, the proportions were 29 of 60 (48%) for ClinVar and 286 of 407 (70%) for MAVE data. Thus, there were more codons appearing to have a singleton DEL variant in ClinVar than on MAVE data.

In silico tools were incorporated into PM5-definitions so that PM5 was not automatically awarded simply because there were colocated DEL variants at the codon, in instances when the variant under examination appeared to be benign. The Grantham Score, Align-GVGD, BLOSUM45, BLOSUM62, and BLOSUM80 reflect the physiochemical difference between wild-type and mutant amino acids. REVEL, Meta-SNP, and CADD are widely-used/high-performing tools, which integrate a wider range of inputs. Overall, all the tools refined the predictive value of PM5, but particular boosting of PM5 pLRs was observed when Meta-SNP, BLOSUM, or The Grantham Score were incorporated into PM5. This is on account of these tools generating lower rates of FP calls. Notably, in this context the tool is used to compare relative deleteriousness between colocated variants, rather than being used with a prespecified binary threshold of pathogenicity, which is more typical in other tool evaluations.^22^^,^^27^

The FP rate is generally low for all PM5-definitions: 3.2% (244/7541) for the most lenient definition of PM5 (PM5-definition_a) and <1% for more stringent PM5-definitions. The low FP rate drives high specificity, positive predictive value, and pLRs for calling of pathogenicity. However, FN rates are high, particularly with increased PM5-definition stringency. Thus, the negative predictive value of PM5 is overall weak and negative likelihood ratios are largely uninformative. Hence, the PM5 metric is only of utility for providing evidence toward pathogenicity and not toward benignity.

Application of PM5 is complicated by pathogenicity due to spliceogenic mechanisms. For the *BRCA1* genomic DNA–based MAVE, spliceogenic DEL variants should give a DEL readout. Interestingly, inclusion of a small number of midexonic potentially spliceogenic variants in *BRCA1* had little effect on PM5 pLRs (Supplemental Table 9). Conversely, for the *MSH2* complementary DNA–based MAVE, spliceogenic DEL variants should not give a DEL readout. A proportion of the ClinVar classifications of P/LP were due to spliceogenic DEL *MSH2* variants; these variants would have inflated the FP rate and dampened the true PM5 pLR for the DEL variants acting via protein effect.

### Limitations

The inherent limitation of these analyses is the use of MAVE-functional classifications as a truthset for pathogenicity. However, although clinical classifications are deemed to be the gold-standard, these are only as good as the comprehensiveness and accuracy of underlying clinical information and the validity of the classification schema employed. Dichotomous classifications are only available in ClinVar for a very modest number of variants; a particular limitation is the very small number of variants for which there is a classification of B/LB. Undeniably, for the *BRCA1/MSH2* variants discrepant between ClinVar and MAVE-functional classification, whereas spliceogenic mechanism of pathogenicity accounted for some of the discrepancies, in several cases, the clinical classification appeared potentially questionable (Supplemental Table 2).^28^^,^^29^ Intermediate penetrance (hypomorphic effect) may also contribute to discrepancies between clinical and functional data.

Although MAVE-functional classifications are unlikely to perfectly recapitulate true human pathogenesis, given their powerful correlation against clinical classifications, the size of the data sets and their systematic generation, arguably represent the best truthsets currently available for this type of large unbiased evaluation of variant classification metrics.

Inherent in PM5 is a presumption of universality across genes that DEL variants will cluster at specific codons that encode functionally important amino acids. However, the extent of gene-by-gene variation in the proportion and tightness of clustering of DEL variants is unclear. Our estimates for *BRCA1* and *MSH2* were overall similar but inclusion of a broader set of genes/MAVEs would be desirable for further exploration of consistency of PM5 pLRs. Although multiple MAVEs were identified for other cancer susceptibility genes *TP53* and *PTEN*, they were not included because of inconsistent correlation between MAVE-functional data sets and the clinical classifications.

### Clinical application

Overall, we would propose on the basis of these analyses that graded evidence levels could be applied for PM5 on the basis of the stringency of PM5 observed. Although incorporation of any of the 8 in silico tools examined improved the magnitude of PM5 discrimination, BLOSUM matrices and The Grantham Score most simply reflect physiochemical protein difference and provide particularly strong discrimination. Using BLOSUM62, PM5 pLR for pathogenicity was found to be 16.3 (10.6-24.9) for a variant under examination for which there is exactly 1 colocated DEL variant and the variant under examination is equally or more damaging (PM5_band_x), and it was found to be 71.5 (37.8-135.3) for a variant under examination for which there are 2 or more colocated DEL variants and the variant under examination is equally or more damaging than at least 1 colocated DEL variant (PM5_band_y). Using the Bayesian formulation of the ACMG/AMP framework proposed by Tavtigian et al,^10^, ^30^ these likelihood ratios would correspond to exponent points of >3 (moderate) and >5 (strong). Even the lower CI would still equate comfortably to >3 points (moderate) and >4 points (strong).^10^^,^^30^^,^^31^ Of note, using BLOSUM62, in total, only 181 of 7541 variants attained PM5: 88 at the lower level (PM5_band_x) and 93 at the higher level (PM5_band_y).

However, careful consideration is required in combining PM5 with evidence items PM1 (hot spot), PS3 (functional data), and PP3 (in silico) to avoid overcounting of nonorthogonal information.^32^ First, we would propose that PM1 should not be used where PM5 is applied; both of these reflect enrichment for pathogenic variants within a prescribed region. Second, we would advocate use of different in silico tools for PM5 and PP3; measures of protein distance such as BLOSUM and The Grantham Score are most apposite for PM5 evaluation, whereas best performance for PP3 is attained by meta tools such as REVEL and Meta-SNP optimized on multiple datasources.^22^ Third, once high quality MAVE data become available for (nearly) all variants in a gene (or region), we would deem that PM5 has been superseded and has become redundant.

## Data Availability

The data analyzed are all publicly available from the references/URLs provided. Although this manuscript does not contain primary research data, materials and data developed during this study will be made available upon request to the corresponding author.

## Conflict of Interest

The authors declare no conflicts of interest.

## Supplementary Material

Supplemental Tables 1 and 4-9

CanVIG-UK Author & Non-Author Contributors List
