# Supplementary material for: Quantifying prediction of pathogenicity for within-codon concordance (PM5) using 7541 functional classifications of BRCA1 and MSH2 missense variants
Source: Genet Med. 2022 Mar;24(3):552–63. doi: 10.1016/j.gim.2021.11.011 (PMC8896276; doi:10.1016/j.gim.2021.11.011)
Supplement: Supplemental Tables 1 and 4-9 [file mmc2.pdf]

Supplementary Tables

Quantifying prediction of pathogenicity for within-codon concordance (PM5) using 7541 functional classifications of BRCA1 and MSH2 missense variants

L.Loong[1*], C. Cubuk[1*], S. Choi[1], S. Allen[1], B.Torr[1], A.Garrett[1], C.Loveday[1], M. Durkie[2], A. Callaway[3, 4], G.J. Burghel[5], J. Drummond[6], R. Robinson[7], I. Berry[7], A. Wallace[5], D. Eccles[8], M. Tischkowitz[9], S.Ellard[10], J. Ware[11, 12], H. Hanson[1, 13], C. Turnbull,[1, 14], and CanVIG-UK**
*These authors contributed equally to this work
**A list of authors and their affiliations appears at the end of the paper

[1] Division of Genetics and Epidemiology, Institute of Cancer Research, Sutton, UK
[2] Sheffield Diagnostic Genetics Service, North East and Yorkshire Genomic Laboratory Hub, Sheffield Children's NHS Foundation Trust, Sheffield, UK
[3] Wessex Regional Genetics Laboratory, Salisbury Hospital NHS Foundation Trust, Salisbury, UK
[4] Human Genetics and Genomic Medicine, Faculty of Medicine, University of Southampton, Southampton, UK
[5] Manchester Centre for Genomic Medicine and NW Laboratory Genetics Hub, Manchester University NHS Foundation Trust, Manchester, UK
[6] East Genomic Laboratory Hub, Cambridge University Hospitals Genomic Laboratory, Cambridge University Hospitals, Cambridge, UK
[7] Yorkshire and North East Genomic Laboratory Hub, Leeds Teaching Hospitals NHS Trust, Leeds, UK
[8] Cancer Sciences, Faculty of Medicine, University of Southampton, Southampton, UK
[9] Department of Medical Genetics, National Institute for Health, Research Cambridge Biomedical Research Centre, University of Cambridge, Cambridge, UK
[10] Department of Molecular Genetics, Royal Devon and Exeter NHS Foundation Trust, Exeter, UK
[11] National Heart and Lung Institute & MRC London Institute of Medical Sciences, Imperial College London, London, UK
[12] Royal Brompton & Harefield Hospitals, London, UK
[13] Department of Clinical Genetics, St. George's University Hospitals NHS Foundation Trust, London, UK
[14] Cancer Genetics Unit, Royal Marsden NHS Foundation Trust, London, UK

Supplementary Table 1: Description of functional assays.
Overview of the functional assay and assay scoring system used for the Findlay et al (*BRCA1*) and the Jia et al (*MSH2*) multiplex assays of variant effect**.**

Supplementary Table 4: Positive likelihood ratios for functional-functional comparison (Additional Approach 1).
Positive likelihood ratios for five definitions of PM5 (a-e) where the functional classification dataset is used to lookup PM5 predictions for the variant-under-examination and the functional classification dataset is used as the reference truthset. (i) *BRCA1* and *MSH2* combined, (ii) *BRCA1*, (iii) *MSH2*.

Supplementary Table 5: Positive likelihood ratios for ClinVar-ClinVar comparison (Additional Approach 2).
Positive likelihood ratios for five definitions of PM5 (a-e) where the clinical classification (ClinVar) dataset is used to lookup PM5 predictions for the variant-under-examination and the clinical classification (ClinVar) dataset is used as the reference truthset. (i) *BRCA1* and *MSH2* combined, (ii) *BRCA1*, (iii) *MSH2*.

Supplementary Table 6: Negative likelihood ratios for different definitions of PM5.
Presented for *BRCA1* and *MSH2* combined. Separated into the main analysis strategy (PM5 predictions made using ClinVar classification dataset and compared to the functional classifications as a truthset), Additional Approach 1 (PM5 predictions: functional dataset. Truthset: functional dataset), and Additional Approach 2 (PM5 predictions: Clinvar dataset. Truthset: ClinVar dataset).

Supplementary Table 7: Positive likelihood ratios for banded analysis for functional-functional comparison (Additional Approach 1).
Positive likelihood ratios for different non-overlapping definitions of PM5 where the functional classification dataset is used to lookup PM5 predictions for the variant-under-examination and the functional classification dataset is used as the reference truthset. (i) *BRCA1* and *MSH2* combined, (ii) *BRCA1*, (iii) *MSH2*.

Supplementary Table 8: Positive likelihood ratios for banded analysis for clinical-clinical comparison (Additional Approach 2).
Positive likelihood ratios for different non-overlapping definitions of PM5 where the ClinVar classification dataset is used to lookup PM5 predictions for the variant-under-examination and the ClinVar classification dataset is used as the reference truthset. (i) *BRCA1* and *MSH2* combined, (ii) *BRCA1*, (iii) *MSH2***.**

Supplementary Table 9: Positive likelihood ratios for main analysis for BRCA1 without and with exclusion of additional spliceogenic variants
31 variants removed, based on quantitative RNAseq: 14 had very low RNA (score<-3; all DEL on functional assay); 17 had low RNA (-3<score<-2; 14 DEL/3 TOL on functional assay)

GENE	Assay type	Assay overview	Assay scoring	Classification 'Tolerated'	Classification 'Deleterious'	Reference
BRCA1	*HAP1 survival assay*	Assay of cellular fitness of HAP1, survival of which is dependent on intact BRCA1 function. Impact on HAP1 survival was examined for 3893 variants in the 13 exons comprising the RING and BRCT functional domains generated via saturation genome editing.	Scores for cell survival were log-normalised across experiments using global medians.	HAP1 functional score >-0.748	HAP1 functional score <-1.328	*Findlay et al 2018*
MSH2	*6-TG resistance assay*	Single amino-acid saturation mutagenesis was applied to generate >17,000 missense variants which encoded nearly all possible amino acid substitutions, synonymous and nonsense variants. These were cloned into a lentiviral vector and transduced into HAP1 cells. Cells were treated with the purine analog 6 thioguanine (6-TG), which is selectively toxic to mismatch repair (MMR) proficient cells, creating lesions that MMR machinary recognises but cannot repair. Quantified abundances of each MSH2 allele before and after 6-TG selection were measured. Functional scores were generated for 16,748 variants.<br><br>For comparability with the BRCA1 data, we only used data for 5734 MSH2 single-nucleotide variants, excluding the variants with 2 or 3 substituted nucleotides.	Median loss of function score taken for each amino acid across three replicates. Score derived from the log2-ratio of variant's frequency after 6-TG treatment divided by its frequency after mock treatment.	Loss of function score <0	Loss of function score >0	*Jia et al, 2021*

Supplementary Table 1

		Additional Approach 1: Functional-PM5-Prediction/Functional-truthset														
		BRCA1+MSH2					BRCA1					MSH2				
**PM5-definition**	**Tool**	**TP**	**FN**	**FP**	**TN**	**positive LR**	**TP**	**FN**	**FP**	**TN**	**positive LR**	**TP**	**FN**	**FP**	**TN**	**positive LR**
PM5_a) ≥1 deleterious reference variants at codon		628	173	1363	5377	**3.9 (3.6-4.1)**	342	52	432	981	**2.8 (2.6-3.1)**	286	121	931	4396	**4.0 (3.7-4.4)**
PM5_b) ≥2 deleterious reference variants at codon		460	341	521	6219	**7.4 (6.7-8.2)**	278	116	210	1203	**4.7 (4.1-5.4)**	182	225	311	5016	**7.7 (6.6-8.9)**
PM5_c) ≥1 deleterious reference variant at codon; variant-under-examination has an equal or more damaging in silico score than ≥1 co-located variant	Revel	460	341	336	6404	**11.5 (10.2-13.0)**	260	134	113	1300	**8.2 (6.8-9.9)**	200	207	223	5104	**11.7 (10.0-13.8)**
	Meta-SNP	453	348	222	6518	**17.1 (14.9-19.8)**	258	136	87	1326	**10.6 (8.5-13.1)**	195	212	135	5192	**18.8 (15.5-22.9)**
	CADD	457	344	429	6311	**9.0 (8.0-10.0)**	256	138	143	1270	**6.4 (5.4-7.6)**	201	206	286	5041	**9.2 (7.9-10.7)**
	Grantham Score	450	351	481	6259	**7.9 (7.1-8.7)**	259	135	144	1269	**6.4 (5.4-7.6)**	191	216	337	4990	**7.4 (6.4-8.6)**
	aGVGD	475	326	395	6345	**10.1 (9.0-11.3)**	260	134	117	1296	**7.9 (6.6-9.6)**	215	192	278	5049	**10.1 (8.7-11.7)**
	Blosum45	534	267	235	6505	**19.1 (16.7-21.8)**	293	101	92	1321	**11.4 (9.2-13.9)**	241	166	143	5184	**22.0 (18.3-26.3)**
	Blosum62	525	276	261	6479	**16.9 (14.8-19.2)**	291	103	103	1310	**10.1 (8.3-12.2)**	234	173	158	5169	**19.3 (16.2-23.0)**
	Blosum80	491	310	255	6485	**16.2 (14.2-18.5)**	272	122	95	1318	**10.2 (8.3-12.5)**	219	188	160	5167	**17.9 (15.0-21.3)**
PM5_d) ≥ 2 deleterious reference variants at codon; variant-under-examination has an equal or more damaging in silico score than ≥ 1 co-located variant	Revel	371	430	139	6601	**22.4 (18.7-26.8)**	225	169	69	1344	**11.6 (9.1-14.8)**	146	261	70	5257	**27.1 (20.8-35.4)**
	Meta-SNP	364	437	85	6655	**35.8 (28.6-44.8)**	225	169	45	1368	**17.7 (13.2-23.9)**	139	268	40	5287	**45.0 (32.2-62.9)**
	CADD	367	434	192	6548	**16.0 (13.7-18.8)**	224	170	76	1337	**10.5 (8.3-13.3)**	143	264	116	5211	**16.1 (12.9-20.1)**
	Grantham Score	364	437	164	6576	**18.6 (15.7-22.1)**	227	167	57	1356	**14.2 (10.8-18.5)**	137	270	107	5220	**16.7 (13.3-21.1)**
	aGVGD	376	425	195	6545	**16.2 (13.8-18.9)**	227	167	57	1356	**14.2 (10.8-18.5)**	149	258	138	5189	**14.1 (11.4-17.4)**
	Blosum45	408	393	98	6642	**34.9 (28.3-42.9)**	245	149	51	1362	**17.1 (12.9-22.6)**	163	244	47	5280	**44.9 (33.1-61.1)**
	Blosum62	409	392	113	6627	**30.3 (25.0-36.8)**	247	147	58	1355	**15.1 (11.7-19.7)**	162	245	55	5272	**38.2 (28.7-51.0)**
	Blosum80	386	415	97	6643	**33.3 (27.0-41.1)**	234	160	50	1363	**16.6 (12.5-22.1)**	152	255	47	5280	**41.9 (30.8-57.1)**
PM5_e) ≥ 2 deleterious reference variants at codon; variant-under-examination has an equal or more damaging in silico score than ≥ 2 co-located variants	Revel	252	549	82	6658	**25.7 (20.3-32.6)**	159	235	34	1379	**16.5 (11.7-23.5)**	93	314	48	5279	**25.2 (18.1-35.1)**
	Meta-SNP	256	545	32	6708	**66.3 (46.4-94.8)**	163	231	21	1392	**27.2 (17.6-42.1)**	93	314	11	5316	**106.2 (58.0-194.3)**
	CADD	263	538	94	6646	**23.4 (18.7-29.3)**	161	233	41	1372	**13.9 (10.1-19.2)**	102	305	53	5274	**25.0 (18.3-34.3)**
	Grantham Score	255	546	53	6687	**40.1 (30.2-53.4)**	163	231	28	1385	**20.5 (14.0-30.1)**	92	315	25	5302	**47.4 (30.9-72.6)**
	aGVGD	267	534	64	6676	**34.9 (26.8-45.3)**	161	233	21	1392	**26.9 (17.4-41.6)**	106	301	43	5284	**32.0 (22.8-44.8)**
	Blosum45	318	483	44	6696	**60.2 (44.3-81.6)**	196	198	19	1394	**36.1 (23.0-56.7)**	122	285	25	5302	**62.7 (41.4-95.0)**
	Blosum62	312	489	50	6690	**52.0 (39.0-69.4)**	188	206	24	1389	**27.5 (18.4-41.3)**	124	283	26	5301	**61.4 (40.8-92.2)**
	Blosum80	307	494	42	6698	**60.8 (44.5-83.1)**	186	208	20	1393	**32.6 (20.9-50.7)**	121	286	22	5305	**70.5 (45.5-109.3)**

Supplementary Table 4

		Additional Approach 2: ClinVar-PM5-Prediction/ClinVar-truthset														
		BRCA1+MSH2					BRCA1					MSH2				
**PM5-definition**	**Tool**	**TP**	**FN**	**FP**	**TN**	**positive LR**	**TP**	**FN**	**FP**	**TN**	**positive LR**	**TP**	**FN**	**FP**	**TN**	**positive LR**
PM5_a) ≥1 deleterious reference variants at codon		91	58	2	48	**12.4 (3.7-42.0)**	62	27	2	20	**6.4 (2.0-20.8)**	29	31	0	28	**28.0 (1.8-443.2)**
PM5_b) ≥2 deleterious reference variants at codon		51	98	2	48	**7.0 (2.0-23.9)**	36	53	2	20	**3.7 (1.1-12.3)**	15	45	0	28	**14.7 (0.9-237.9)**
PM5_c) ≥1 deleterious reference variant at codon; variant-under-examination has an equal or more damaging in silico score than ≥1 co-located variant	Revel	58	91	0	50	**39.8 (2.5-631.9)**	40	49	0	22	**20.7 (1.3-324.1)**	18	42	0	28	**17.6 (1.1-281.8)**
	Meta-SNP	58	91	0	50	**39.8 (2.5-631.9)**	40	49	0	22	**20.7 (1.3-324.1)**	18	42	0	28	**17.6 (1.1-281.8)**
	CADD	61	88	1	49	**13.9 (2.8-68.2)**	42	47	1	21	**7.2 (1.5-34.5)**	19	41	0	28	**18.5 (1.2-296.5)**
	Grantham Score	59	90	0	50	**40.5 (2.5-642.6)**	41	48	0	22	**21.2 (1.4-332.0)**	18	42	0	28	**17.6 (1.1-281.8)**
	aGVGD	63	86	0	50	**43.2 (2.7-685.3)**	41	48	0	22	**21.2 (1.4-332.0)**	22	38	0	28	**21.4 (1.3-340.5)**
	Blosum45	72	77	0	50	**49.3 (3.1-781.4)**	50	39	0	22	**25.8 (1.7-402.8)**	22	38	0	28	**21.4 (1.3-340.5)**
	Blosum62	73	76	0	50	**50.0 (3.2-792.1)**	49	40	0	22	**25.3 (1.6-394.9)**	24	36	0	28	**23.3 (1.5-369.8)**
	Blosum80	68	81	0	50	**46.6 (2.9-738.7)**	44	45	0	22	**22.7 (1.5-355.6)**	24	36	0	28	**23.3 (1.5-369.8)**
PM5_d) ≥ 2 deleterious reference variants at codon; variant-under-examination has an equal or more damaging in silico score than ≥ 1 co-located variant	Revel	37	112	0	50	**25.5 (1.6-407.8)**	27	62	0	22	**14.1 (0.9-221.9)**	10	50	0	28	**10.0 (0.6-164.6)**
	Meta-SNP	37	112	0	50	**25.5 (1.6-407.8)**	27	62	0	22	**14.1 (0.9-221.9)**	10	50	0	28	**10.0 (0.6-164.6)**
	CADD	39	110	1	49	**9.0 (1.8-44.3)**	29	60	1	21	**5.0 (1.0-24.3)**	10	50	0	28	**10.0 (0.6-164.6)**
	Grantham Score	38	111	0	50	**26.2 (1.6-418.5)**	28	61	0	22	**14.6 (0.9-229.8)**	10	50	0	28	**10.0 (0.6-164.6)**
	aGVGD	38	111	0	50	**26.2 (1.6-418.5)**	28	61	0	22	**14.6 (0.9-229.8)**	10	50	0	28	**10.0 (0.6-164.6)**
	Blosum45	42	107	0	50	**28.9 (1.8-461.2)**	30	59	0	22	**15.6 (1.0-245.5)**	12	48	0	28	**11.9 (0.7-193.9)**
	Blosum62	44	105	0	50	**30.3 (1.9-482.5)**	31	58	0	22	**16.1 (1.0-253.3)**	13	47	0	28	**12.8 (0.8-208.5)**
	Blosum80	43	106	0	50	**29.6 (1.9-471.8)**	30	59	0	22	**15.6 (1.0-245.5)**	13	47	0	28	**12.8 (0.8-208.5)**
PM5_e) ≥ 2 deleterious reference variants at codon; variant-under-examination has an equal or more damaging in silico score than ≥ 2 co-located variants	Revel	26	123	0	50	**18.0 (1.1-290.4)**	20	69	0	22	**10.5 (0.7-166.8)**	6	54	0	28	**6.2 (0.4-106.0)**
	Meta-SNP	30	119	0	50	**20.7 (1.3-333.1)**	22	67	0	22	**11.5 (0.7-182.6)**	8	52	0	28	**8.1 (0.5-135.3)**
	CADD	27	122	1	49	**6.2 (1.2-31.3)**	20	69	1	21	**3.5 (0.7-17.2)**	7	53	0	28	**7.1 (0.4-120.6)**
	Grantham Score	26	123	0	50	**18.0 (1.1-290.4)**	20	69	0	22	**10.5 (0.7-166.8)**	6	54	0	28	**6.2 (0.4-106.0)**
	aGVGD	26	123	0	50	**18.0 (1.1-290.4)**	20	69	0	22	**10.5 (0.7-166.8)**	6	54	0	28	**6.2 (0.4-106.0)**
	Blosum45	34	115	0	50	**23.5 (1.5-375.8)**	28	61	0	22	**14.6 (0.9-229.8)**	6	54	0	28	**6.2 (0.4-106.0)**
	Blosum62	31	118	0	50	**21.4 (1.3-343.8)**	26	63	0	22	**13.5 (0.9-214.0)**	5	55	0	28	**5.2 (0.3-91.4)**
	Blosum80	31	118	0	50	**21.4 (1.3-343.8)**	26	63	0	22	**13.5 (0.9-214.0)**	5	55	0	28	**5.2 (0.3-91.4)**

Supplementary Table 5

PM5-definition	Tool	Main analysis: ClinVar-PM5-Prediction/Functional-truth-set					Additional Approach 1: Functional-PM5-Prediction/Functional-truth-set					Additional Approach 2: ClinVar-PM5-Prediction/ClinVar-truth-set				
		**TP**	**FN**	**FP**	**TN**	**negative LR**	**TP**	**FN**	**FP**	**TN**	**negative LR**	**TP**	**FN**	**FP**	**TN**	**negative LR**
PM5_a) ≥1 deleterious reference variants at codon		245	556	244	6496	**1.4 (1.3-1.5)**	628	173	1363	5377	**3.7 (3.2-4.2)**	91	58	2	48	**2.4 (2.0-3.0)**
PM5_b) ≥2 deleterious reference variants at codon		111	690	59	6681	**1.2 (1.1-1.2)**	460	341	521	6219	**2.2 (2.0-2.3)**	51	98	2	48	**1.4 (1.3-1.7)**
PM5_c) ≥1 deleterious reference variant at codon; variant-under-examination has an equal or more damaging in silico score than ≥1 co-located variant	Revel	122	679	52	6688	**1.2 (1.1-1.2)**	460	341	336	6404	**2.2 (2.1-2.4)**	58	91	0	50	**1.6 (1.4-1.9)**
	Meta-SNP	145	656	29	6711	**1.2 (1.2-1.3)**	453	348	222	6518	**2.2 (2.1-2.4)**	58	91	0	50	**1.6 (1.4-1.9)**
	CADD	154	647	77	6663	**1.2 (1.2-1.3)**	457	344	429	6311	**2.2 (2.0-2.4)**	61	88	1	49	**1.6 (1.4-1.9)**
	Grantham Score	142	659	50	6690	**1.2 (1.2-1.2)**	450	351	481	6259	**2.1 (2.0-2.3)**	59	90	0	50	**1.6 (1.4-1.9)**
	aGVGD	141	660	54	6686	**1.2 (1.2-1.2)**	475	326	395	6345	**2.3 (2.1-2.5)**	63	86	0	50	**1.7 (1.5-2.0)**
	Blosum45	133	668	40	6700	**1.2 (1.2-1.2)**	534	267	235	6505	**2.9 (2.6-3.2)**	72	77	0	50	**1.9 (1.6-2.2)**
	Blosum62	139	662	42	6698	**1.2 (1.2-1.2)**	525	276	261	6479	**2.8 (2.5-3.1)**	73	76	0	50	**1.9 (1.7-2.3)**
	Blosum80	133	668	43	6697	**1.2 (1.2-1.2)**	491	310	255	6485	**2.5 (2.3-2.7)**	68	81	0	50	**1.8 (1.6-2.1)**
PM5_d) ≥ 2 deleterious reference variants at codon; variant-under-examination has an equal or more damaging in silico score than ≥ 1 co-located variant	Revel	69	732	15	6725	**1.1 (1.1-1.1)**	371	430	139	6601	**1.8 (1.7-1.9)**	37	112	0	50	**1.3 (1.2-1.5)**
	Meta-SNP	81	720	6	6734	**1.1 (1.1-1.1)**	364	437	85	6655	**1.8 (1.7-1.9)**	37	112	0	50	**1.3 (1.2-1.5)**
	CADD	86	715	28	6712	**1.1 (1.1-1.1)**	367	434	192	6548	**1.8 (1.7-1.9)**	39	110	1	49	**1.3 (1.2-1.5)**
	Grantham Score	73	728	5	6735	**1.1 (1.1-1.1)**	364	437	164	6576	**1.8 (1.7-1.9)**	38	111	0	50	**1.3 (1.2-1.5)**
	aGVGD	72	729	19	6721	**1.1 (1.1-1.1)**	376	425	195	6545	**1.8 (1.7-2.0)**	38	111	0	50	**1.3 (1.2-1.5)**
	Blosum45	77	724	9	6731	**1.1 (1.1-1.1)**	408	393	98	6642	**2.0 (1.9-2.2)**	42	107	0	50	**1.4 (1.2-1.5)**
	Blosum62	83	718	10	6730	**1.1 (1.1-1.1)**	409	392	113	6627	**2.0 (1.9-2.2)**	44	105	0	50	**1.4 (1.3-1.6)**
	Blosum80	79	722	9	6731	**1.1 (1.1-1.1)**	386	415	97	6643	**1.9 (1.8-2.0)**	43	106	0	50	**1.4 (1.3-1.6)**
PM5_e) ≥ 2 deleterious reference variants at codon; variant-under-examination has an equal or more damaging in silico score than ≥ 2 co-located variants	Revel	42	759	7	6733	**1.1 (1.0-1.1)**	252	549	82	6658	**1.4 (1.4-1.5)**	26	123	0	50	**1.2 (1.1-1.3)**
	Meta-SNP	49	752	3	6737	**1.1 (1.0-1.1)**	256	545	32	6708	**1.5 (1.4-1.5)**	30	119	0	50	**1.2 (1.1-1.4)**
	CADD	52	749	17	6723	**1.1 (1.0-1.1)**	263	538	94	6646	**1.5 (1.4-1.5)**	27	122	1	49	**1.2 (1.1-1.3)**
	Grantham Score	44	757	1	6739	**1.1 (1.0-1.1)**	255	546	53	6687	**1.5 (1.4-1.5)**	26	123	0	50	**1.2 (1.1-1.3)**
	aGVGD	43	758	3	6737	**1.1 (1.0-1.1)**	267	534	64	6676	**1.5 (1.4-1.6)**	26	123	0	50	**1.2 (1.1-1.3)**
	Blosum45	49	752	4	6736	**1.1 (1.0-1.1)**	318	483	44	6696	**1.6 (1.6-1.7)**	34	115	0	50	**1.3 (1.2-1.4)**
	Blosum62	43	758	5	6735	**1.1 (1.0-1.1)**	312	489	50	6690	**1.6 (1.5-1.7)**	31	118	0	50	**1.3 (1.1-1.4)**
	Blosum80	46	755	6	6734	**1.1 (1.0-1.1)**	307	494	42	6698	**1.6 (1.5-1.7)**	31	118	0	50	**1.3 (1.1-1.4)**

Supplementary Table 6

PM5-definition	Tool	Additional Approach 1: Functional-PM5-Prediction/Functional-truth-set														
		BRCA1+MSH2					BRCA1					MSH2				
		**TP**	**FN**	**FP**	**TN**	**positive LR**	**TP**	**FN**	**FP**	**TN**	**positive LR**	**TP**	**FN**	**FP**	**TN**	**positive LR**
PM5_baseline_band: variants not attaining criteria for PM5_band_x or PM5_band_y																
PM5_band_x) Exactly 1 deleterious co-located variant at codon; variant-under-examination has an equal or more damaging in silico score than co-located variant; comparison to baseline variant set	Revel	89	341	197	6404	**6.9 (5.5-8.7)**	35	134	44	1300	**6.3 (4.2-9.5)**	54	207	153	5104	**7.1 (5.4-9.5)**
	Meta-SNP	89	348	137	6518	**9.9 (7.7-12.7)**	33	136	42	1326	**6.3 (4.2-9.7)**	56	212	95	5192	**11.6 (8.6-15.8)**
	CADD	90	344	237	6311	**5.7 (4.6-7.2)**	32	138	67	1270	**3.8 (2.6-5.5)**	58	206	170	5041	**6.7 (5.2-8.8)**
	Grantham Score	86	351	317	6259	**4.1 (3.3-5.1)**	32	135	87	1269	**3.0 (2.1-4.3)**	54	216	230	4990	**4.6 (3.5-6.0)**
	aGVGD	99	326	200	6345	**7.6 (6.1-9.5)**	33	134	60	1296	**4.5 (3.0-6.6)**	66	192	140	5049	**9.5 (7.3-12.3)**
	Blosum45	126	267	137	6505	**15.5 (12.5-19.3)**	48	101	41	1321	**10.6 (7.3-15.5)**	78	166	96	5184	**17.5 (13.4-22.9)**
	Blosum62	116	276	148	6479	**13.2 (10.6-16.5)**	44	103	45	1310	**9.0 (6.1-13.1)**	72	173	103	5169	**15.0 (11.4-19.7)**
	Blosum80	105	310	158	6485	**10.6 (8.5-13.3)**	38	122	45	1318	**7.2 (4.8-10.7)**	67	188	113	5167	**12.3 (9.3-16.1)**
PM5_band_y) ≥2 deleterious co-located variants at codon; variant-under-examination has an equal or more damaging in silico score than ≥1 co-located variant; comparison to baseline variant set	Revel	371	341	139	6404	**24.4 (20.4-29.2)**	225	134	69	1300	**12.3 (9.7-15.7)**	146	207	70	5104	**30.4 (23.4-39.5)**
	Meta-SNP	364	348	85	6518	**39.5 (31.6-49.3)**	225	136	45	1326	**18.8 (14.0-25.3)**	139	212	40	5192	**51.2 (36.7-71.4)**
	CADD	367	344	192	6311	**17.4 (14.9-20.4)**	224	138	76	1270	**10.9 (8.6-13.7)**	143	206	116	5041	**18.2 (14.6-22.6)**
	Grantham Score	367	344	192	6311	**17.4 (14.9-20.4)**	227	135	57	1269	**14.5 (11.1-18.9)**	137	216	107	4990	**18.4 (14.7-23.1)**
	aGVGD	376	326	195	6345	**17.9 (15.4-20.9)**	227	134	57	1296	**14.8 (11.4-19.3)**	149	192	138	5049	**16.4 (13.4-20.1)**
	Blosum45	408	267	98	6505	**40.5 (33.0-49.7)**	245	101	51	1321	**18.9 (14.3-24.9)**	163	166	47	5184	**54.6 (40.3-73.9)**
	Blosum62	409	276	113	6479	**34.7 (28.6-42.0)**	247	103	58	1310	**16.5 (12.7-21.4)**	162	173	55	5169	**45.5 (34.3-60.5)**
	Blosum80	386	310	97	6485	**37.4 (30.4-46.1)**	234	122	50	1318	**17.8 (13.4-23.6)**	152	188	47	5167	**49.1 (36.1-66.7)**

Supplementary Table 7

PM5-definition	Tool	Additional Approach 2: ClinVar-PM5-Prediction/ClinVar-truth-set														
		BRCA1+MSH2					BRCA1					MSH2				
		**TP**	**FN**	**FP**	**TN**	**positive LR**	**TP**	**FN**	**FP**	**TN**	**positive LR**	**TP**	**FN**	**FP**	**TN**	**positive LR**
PM5_baseline_band: variants not attaining criteria for PM5_band_x or PM5_band_y																
PM5_band_x) Exactly 1 deleterious co-located variant at codon; variant-under-examination has an equal or more damaging in silico score than co-located variant; comparison to baseline variant set	Revel	21	91	0	50	**19.4 (1.2-314.2)**	13	49	0	22	**9.9 (0.6-159.2)**	8	42	0	28	**9.7 (0.6-161.4)**
	Meta-SNP	21	91	0	50	**19.4 (1.2-314.2)**	13	49	0	22	**9.9 (0.6-159.2)**	8	42	0	28	**9.7 (0.6-161.4)**
	CADD	22	88	0	49	**20.3 (1.3-327.5)**	13	47	0	21	**9.7 (0.6-157.0)**	9	41	0	28	**10.8 (0.7-178.9)**
	Grantham Score	21	90	0	50	**19.6 (1.2-317.0)**	13	48	0	22	**10.0 (0.6-161.7)**	8	42	0	28	**9.7 (0.6-161.4)**
	aGVGD	25	86	0	50	**23.2 (1.4-374.0)**	13	48	0	22	**10.0 (0.6-161.7)**	12	38	0	28	**14.2 (0.9-231.4)**
	Blosum45	30	77	0	50	**28.8 (1.8-461.8)**	20	39	0	22	**15.7 (1.0-249.3)**	10	38	0	28	**12.4 (0.8-204.3)**
	Blosum62	29	76	0	50	**28.4 (1.8-455.4)**	18	40	0	22	**14.4 (0.9-229.6)**	11	36	0	28	**13.9 (0.9-227.1)**
	Blosum80	25	81	0	50	**24.3 (1.5-391.4)**	14	45	0	22	**11.1 (0.7-178.8)**	11	36	0	28	**13.9 (0.9-227.1)**
PM5_band_y) ≥2 deleterious co-located variants at codon; variant-under-examination has an equal or more damaging in silico score than ≥1 co-located variant; comparison to baseline variant set	Revel	37	91	0	50	**29.7 (1.9-473.8)**	27	49	0	22	**16.4 (1.0-259.0)**	10	42	0	28	**11.5 (0.7-189.1)**
	Meta-SNP	37	91	0	50	**29.7 (1.9-473.8)**	27	49	0	22	**16.4 (1.0-259.0)**	10	42	0	28	**11.5 (0.7-189.1)**
	CADD	39	88	1	49	**10.5 (2.1-51.9)**	29	47	1	21	**5.9 (1.2-28.3)**	10	41	0	28	**11.7 (0.7-192.7)**
	Grantham Score	39	88	1	49	**10.5 (2.1-51.9)**	28	48	0	22	**17.0 (1.1-268.2)**	10	42	0	28	**11.5 (0.7-189.1)**
	aGVGD	38	86	0	50	**31.4 (2.0-501.7)**	28	48	0	22	**17.0 (1.1-268.2)**	10	38	0	28	**12.4 (0.8-204.3)**
	Blosum45	42	77	0	50	**36.1 (2.3-575.8)**	30	39	0	22	**20.0 (1.3-314.9)**	12	38	0	28	**14.2 (0.9-231.4)**
	Blosum62	44	76	0	50	**37.5 (2.4-597.5)**	31	40	0	22	**20.1 (1.3-316.1)**	13	36	0	28	**15.7 (1.0-253.8)**
	Blosum80	43	81	0	50	**35.5 (2.2-565.7)**	30	45	0	22	**18.5 (1.2-290.3)**	13	36	0	28	**15.7 (1.0-253.8)**

Supplementary Table 8

PM5-definition	Tool	BRCA1: main analysis					BRCA1: main analysis with removal of 31 additional spliceogenic variants				
		TP	FN	FP	TN	positive LR	TP	FN	FP	TN	positive LR
PM5_a) ≥1 deleterious reference variants at codon		161	233	97	1316	**5.9 (4.7-7.4)**	150	216	79	1331	**7.3 (5.7-9.3)**
PM5_b) ≥2 deleterious reference variants at codon		84	310	29	1384	**10.3 (6.8-15.4)**	79	287	28	1382	**10.7 (7.1-16.2)**
PM5_c) ≥1 deleterious reference variant at codon; variant-under-examination has an equal or more damaging in silico score than ≥1 co-located variant	Revel	84	310	15	1398	**19.5 (11.5-33.2)**	80	286	10	1400	**29.5 (15.7-55.5)**
	Meta-SNP	99	295	9	1404	**37.5 (19.5-72.3)**	91	275	6	1404	**54.1 (24.6-118.9)**
	CADD	109	285	37	1376	**10.5 (7.3-14.9)**	100	266	27	1383	**14.1 (9.4-21.1)**
	Grantham Score	90	304	20	1393	**15.8 (9.9-25.2)**	84	282	14	1396	**22.4 (13.0-38.6)**
	aGVGD	90	304	14	1399	**22.3 (13.0-38.5)**	84	282	11	1399	**28.3 (15.4-51.7)**
	Blosum45	91	303	12	1401	**26.2 (14.7-46.8)**	86	280	8	1402	**39.1 (19.5-78.4)**
	Blosum62	93	301	12	1401	**26.8 (15.0-47.8)**	88	278	8	1402	**40.0 (20.0-80.2)**
	Blosum80	87	307	12	1401	**25.1 (14.0-44.8)**	84	282	7	1403	**43.3 (20.7-90.6)**
PM5_d) ≥ 2 deleterious reference variants at codon; variant-under-examination has an equal or more damaging in silico score than ≥ 1 co-located variant	Revel	53	341	4	1409	**42.6 (16.4-110.7)**	50	316	4	1406	**43.1 (16.6-112.4)**
	Meta-SNP	62	332	4	1409	**49.7 (19.2-128.6)**	57	309	3	1407	**63.2 (21.6-184.7)**
	CADD	68	326	16	1397	**14.9 (8.8-25.1)**	63	303	16	1394	**14.8 (8.7-25.1)**
	Grantham Score	52	342	2	1411	**75.2 (21.2-266.0)**	50	316	2	1408	**77.7 (21.9-275.1)**
	aGVGD	52	342	2	1411	**75.2 (21.2-266.0)**	50	316	2	1408	**77.7 (21.9-275.1)**
	Blosum45	58	336	4	1409	**46.5 (18.0-120.6)**	55	311	4	1406	**47.4 (18.3-123.1)**
	Blosum62	61	333	4	1409	**48.9 (18.9-126.6)**	58	308	4	1406	**50.0 (19.3-129.5)**
	Blosum80	59	335	3	1410	**60.9 (20.8-177.8)**	56	310	3	1407	**62.1 (21.2-181.6)**
PM5_e) ≥ 2 deleterious reference variants at codon; variant-under-examination has an equal or more damaging in silico score than ≥ 2 co-located variants	Revel	34	360	1	1412	**82.3 (16.1-420.6)**	31	335	1	1409	**80.7 (15.8-413.7)**
	Meta-SNP	36	358	2	1411	**52.3 (14.6-187.3)**	33	333	2	1408	**51.5 (14.3-185.3)**
	CADD	43	351	11	1402	**13.5 (7.1-25.7)**	41	325	11	1399	**13.9 (7.3-26.4)**
	Grantham Score	34	360	0	1413	**247.0 (15.2-4,019.8)**	33	333	0	1410	**257.6 (15.8-4,194.1)**
	aGVGD	34	360	0	1413	**247.0 (15.2-4,019.8)**	33	333	0	1410	**257.6 (15.8-4,194.1)**
	Blosum45	41	353	0	1413	**297.1 (18.3-4,819.2)**	40	326	0	1410	**311.4 (19.2-5,052.5)**
	Blosum62	37	357	0	1413	**268.5 (16.5-4,362.4)**	36	330	0	1410	**280.7 (17.3-4,562.0)**
	Blosum80	39	355	1	1412	**94.3 (18.5-479.5)**	37	329	1	1409	**96.1 (18.9-489.6)**

Supplementary Table 9
