# Supplementary material for: Quantifying prediction of pathogenicity for within-codon concordance (PM5) using 7541 functional classifications of BRCA1 and MSH2 missense variants
Source: Genet Med. 2022 Mar;24(3):552–63. doi: 10.1016/j.gim.2021.11.011 (PMC8896276; doi:10.1016/j.gim.2021.11.011)
Supplement: CanVIG-UK Author & Non-Author Contributors List [file mmc3.pdf]

SUPPLEMENTARY NOTE

CANVIG-UK

AUTHORS

Lucy Loong[1], Alice Garrett[1], Subin Choi[1], Beth Torr[1], Sophie Allen[1], Miranda Durkie[2], Alison Callaway[3], James Drummond[4], George J Burghel[5], Rachel Robinson[6], Ian R Berry[65], Andrew J Wallace[5], Diana M Eccles[7, 8], Marc Tischkowitz[13], Sian Ellard[9], Helen Hanson[1,16], and Clare Turnbull[1,49]

CANVIG-UK

NON-AUTHOR CONTRIBUTORS

Emma Baple[10, 11], D Gareth Evans[5, 11], Emma Woodward[5], Fiona Lalloo[5], Anneke Lucassen[14, 15]S. Samant[33] , A. Lucassen[57] , A. Znaczko[44], A. Shaw[23] , A. Ansari[34] , A. Kumar[21], A. Donaldson[53] , A. Murray[19] , A. Ross[18] , A. Taylor-Beadling[22] , A. Taylor[18] , A. Innes[25] , A. Brady[29] , A. Kulkarni[23] , A.-C. Hogg[5] , A. Ramsay Bowden[18] , A. Hadonou[47] , B. Coad[16] , B. McIldowie[19] , B. Speight[18] , B. DeSouza[47] , B. Mullaney[3] , C. McKenna[62] , C. Brewer[44] , C. Olimpio[18] , C. Clabby[40] , C. Crosby[47] , C. Jenkins[42] , C. Armstrong[33] , C. Bowles[9] , C. Brooks[22] , C. Byrne[62] , C. Maurer[4] , D. Baralle[57] , D. Chubb[1] , D. Stobo[34] , D. Moore[35] , D. O'Sullivan[33] , D. Donnelly[62] , D. Randhawa[24] , D. Halliday[41] , E. Atkinson[50] , E. Baple[20] , E. Rauter[24] , E. Johnston[38] , E. Woodward[2,30] , E. Maher[8], E. Sofianopoulou[17] , E. Petrides[42] , F. Lalloo[2] , F. McRonald[43] , F. Pelz[51] , I. Frayling[19] , G. Evans[2,30] , G. Corbett[62] , G. Rea[62] , H. Clouston[5] , H. Powell[31] , H. Williamson[52] , H. Carley[47] , H.J.W. Thomas[26], I. Tomlinson[63] , J. Cook[46] , J. Hoyle[21] , J. Tellez[32] , J. Whitworth[18] , J. Williams[49] , J. Murray[35] , J. Campbell[27] , J. Tolmie[33] , J. Field[38] , J. Mason[64] , J. Burn[31] , J. Bruty[18] , J. Callaway[8] , J. Grant[34] , J. Del Rey Jimenez[47] , J. Pagan[35] , J. VanCampen[24] , J. Barwell[53] , K. Monahan[29] , K. Tatton-Brown[16] , K.-R. Ong[63] , K. Murphy[33] , K. Andrews[18] , K. Mokretar[23] , K. Cadoo[48] , K. Smith[52] , K. Baker[8] , K. Brown[24] , K. Reay[64] ,K. McKay Bounford[34] , K. Bradshaw[38] , K. Russell[65] , K. Stone[23] , K. Snape[16] , L. Crookes[5] , L. Reed[21] , L. Taggart[62] , L. Yarram[65] , L. Cobbold[47] , L. Walker[39] , L. Walker[41] , L. Hawkes[16] , L. Busby[22] , L. Izatt[23] , L. Kiely[22] , L. Hughes[64] , L. Side[56] , L. Sarkies[18] , K.-L. Greenhalgh[28], M. Shanmugasundaram[63] , M. Duff[40] , M. Bartlett[29] , M. Watson[3] , M. Owens[9] , M. Bradford[54] , M. Huxley[64] , M. Slean[33] , M. Ryten[23] , M. Smith[55] , M. Ahmed[21] , N. Roberts[2] , C. O'Brien[50] , O. Middleton[33] , P. Tarpey[4] , P. Logan[62] , P. Dean[3] , P. May[24] , P. Brace[21] , R. Tredwell[38] , R. Harrison[37] , R. Hart[63] , R. Kirk[5] , R. Martin[31] , R. Nyanhete[3] , R. Wright[2], R. Martin[62] , R. Davidson[34] , R. Cleaver[45] , S. Talukdar[16], S. Butler[64] , J. Sampson[19] , S. Ribeiro[49] , S. Dell[46] , S. Mackenzie[32] , S. Hegarty[62] , S. Albaba[5] , S. McKee[36] , S. Palmer-Smith[19] , S. Heggarty[62], S. MacParland[62] , S. Greville-Heygate[58] , S. Daniels[4] ,S. Prapa[18] ,S. Abbs[4] , S. Tennant[33] , S. Hardy[43] , S. MacMahon[49] , T. McVeigh[49], T. Foo[49] , T. Bedenham[42] , T. Cranston[42] , T. McDevitt[40] , V. Clowes[29] , V. Tripathi[23] , V. McConnell[62] , N. Woodwaer[45] , Y. Wallis[64] , Z. Kemp[49], G. Mullan[62] , L. Pierson[62], L. Rainey[62], C. Joyce[59], A. Timbs[41], A-M. Reuther[3], B. Frugtniet[19], B. DeSouza[25], C. Husher[3], C. Lawn[22], C. Corbett[63], D. Nocera-Jijon[16], D. Reay[31], E. Cross[3], F. Ryan[3], H. Lindsay[6], J. Oliver[6], J. Dring[63], J. Spiers[65], J. Harper[23], K. Ciucias[34], L. Connolly[60], M. Tsang[62], R. Brown[6], S. Shepherd[32], S. Begum[16], S. Daniels[3], T. Tadiso[16], T. Linton-Willoughby[16], H. Heppell[35], K. Sahan[61], L. Worrillow[6], Z. Allen[22], M. Barlett[29], C. Watt[34],M. Hegarty[62], R. Mitchell[6], R. Coles[66], G. Nickless[23], E. Cojocaru[49], I. Doal[64], and F. Sava[64]

1 Division of Genetics and Epidemiology, Institute of Cancer Research, Sutton, UK

2 Yorkshire and North East Genomic Laboratory Hub, Sheffield Children's NHS Foundation Trust, Sheffield, UK

3 Central and South Genomics Laboratory Hub, Wessex Regional Genetics Laboratory, Salisbury NHS Foundation Trust, Salisbury, UK

4 East Genomic Laboratory Hub, Cambridge University Hospitals Genomic Laboratory, Cambridge University NHS Foundation Trust, Cambridge, UK

5 Manchester Centre for Genomic Medicine and North West Genomic Laboratory Hub, Manchester University NHS Foundation Trust, Manchester, UK

6 Yorkshire and North East Genomic Laboratory Hub, Leeds Teaching Hospitals NHS Trust, Leeds, UK

7 Cancer Sciences, Faculty of Medicine, University of Southampton, Southampton, UK

8 Human Genetics and Genomic Medicine, Faculty of Medicine, University of Southampton, Southampton, UK

9 Department of Molecular Genetics, Royal Devon and Exeter NHS Foundation Trust, Exeter, UK

10 Genomics England, London, UK

11 University of Exeter Medical School, Exeter, UK

12 Division of Evolution & Genomic Sciences, The University of Manchester

13 Department of Medical Genetics, National Institute for Health, Research Cambridge Biomedical Research Centre, University of Cambridge, Cambridge, UK

14 Wessex Clinical Genetics Service, University Hospital Southampton NHS Foundation Trust, Southampton, UK

15 Clinical Ethics and Law Unit, University of Southampton, Southampton, UK

16 Department of Clinical Genetics, St. George's University Hospitals NHS Foundation Trust, London, UK

[17] Public Health and Primary Care, Clinical Medicine, University of Cambridge, Cambridge, UK

[18] Cambridge University Hospitals NHS Foundation Trust, Cambridge, UK

[19] Institute of Medical Genetics, University Hospital of Wales, Cardiff and Vale University Health Board, Cardiff, UK

[20] Genomics England, London, UK

[21] Great Ormond Street Hospital for Children NHS Foundation Trust, London, UK

[22] North Thames Genomic Laboratory Hub, Great Ormond Street Hospital for Children NHS Foundation Trust, London, UK

[23] Department of Clinical Genetics, Guy’s and St Thomas’ NHS Foundation Trust, London, UK

[24] South East Genomic Laboratory Hub, Guy's and St Thomas' NHS Foundation Trust, London, UK

[25] Genomic Medicine Service, Imperial College Healthcare NHS Trust, London, UK

[26] Faculty of Medicine, Department of Surgery & Cancer, Imperial College London, London, UK

[27] Institute of Neurology, UCL Queen Square Institute of Neurology, London, UK

[28] Liverpool Women's NHS Foundation Trust, Liverpool, UK

[29] London North West University Healthcare NHS Trust, London, UK

[30] Division of Evolution and Genomic Sciences, School of Biological Sciences, Faculty of Biology Medicine and Health, The University of Manchester, Manchester, UK.

[31] The Newcastle upon Tyne Hospitals NHS Foundation Trust, Newcastle upon Tyne, UK

[32] North East and Yorkshire Genomic Laboratory Hub, The Newcastle upon Tyne Hospitals NHS Foundation Trust, Newcastle upon Tyne, UK

[33] NHS Grampian, Aberdeen, UK

[34] NHS Greater Glasgow and Clyde, Glasgow, UK

[35] NHS Lothian, Edinburgh, UK

[36] Northern Ireland Regional Genetics Service, Belfast Health & Social Care Trust, Belfast, UK

[37] Nottingham University Hospitals NHS Trust, Nottingham, UK

[38] East Midlands and East of England Genomics Laboratory, Nottingham University Hospitals NHS Trust, Nottingham, UK

[39] University of Otago, Otago, New Zealand

[40] Our Lady's Children's Hospital, Crumlin, Dublin, Ireland

[41] Clinical Genetics, Oxford University Hospitals NHS Foundation Trust, Oxford, UK

[42] West Midlands, Oxford and Wessex Genomic Laboratory Hub, Oxford University Hospitals NHS Foundation Trust, Oxford, UK

[43] Public Health England, London, UK

[44] Royal Devon and Exeter NHS Foundation Trust, Exeter, UK

[45] Royal Free London NHS Foundation Trust, London, UK

[46] Sheffield Children's NHS Foundation Trust, Sheffield, UK

[47] South East Genomics Laboratory Hub, St George's University Hospitals NHS Foundation Trust, London, UK

[48] St James's Hospital, Dublin, Ireland

[49] Cancer Genetics Unit, The Royal Marsden NHS Foundation Trust, Sutton, London, UK

[50] Trinity College Dublin, The University of Dublin, Ireland

[51] University Hospital of Wales, Cardiff and Vale University Health Board, Cardiff, UK

[52] University Hospitals Bristol NHS Foundation Trust, Bristol, UK

[53] University Hospitals of Leicester NHS Trust, Leicester, UK

[54] University Hospitals of Plymouth NHS Trust, Plymouth, UK

[55] University of Manchester, Manchester, UK

[56] Wessex Clinical Genetics Service, Princess Anne Hospital, Southampton, UK

[57] Faculty of Medicine, University of Southampton, Southampton, UK

[58] University Hospital Southampton NHS Foundation Trust, Southampton, UK

[59] Cork University Hospital, Cork, Ireland

[60] Children's Health Ireland (CHI), Crumlin, Dublin, Ireland

[61] The Ethox Centre, Oxford, UK

[62] Belfast Health & Social Care Trust, Belfast, UK

[63] Birmingham Women's and Children's NHS Foundation Trust, Birmingham, UK

[64] Central and South Genomic Laboratory Hub, Birmingham Women's and Children's NHS Foundation Trust, Birmingham, UK

[65] Bristol Genetics Laboratory, North Bristol NHS Trust, Bristol, UK

[66] Northwick Park Hospital, Watford Rd, Harrow HA1 3UJ
